# Spatial biogeography of microbes in soils vs. aquatic ecosystems in U.S.’s major natural biomes

**DOI:** 10.3389/fmicb.2026.1752205

**Published:** 2026-03-12

**Authors:** Leo Oo Sai, Xinhao Zhu, David A. Lipson, Xiaofeng Xu

**Affiliations:** Biology Department, San Diego State University, San Diego, CA, United States

**Keywords:** biogeography, cell density, microbial biomass, soil, water

## Abstract

**Introduction:**

Microbial macroecology has gained recognition as a critical component of microbial ecology. Using data from the National Ecological Observatory Network (NEON), this study examined the spatial patterns of microbial abundance and their environmental controls in soil and aquatic ecosystems across major natural biomes in the United States.

**Methods:**

Microbial cell density in aquatic ecosystems and soil microbial biomass carbon in terrestrial ecosystems were analyzed, and their relationships with environmental variables were evaluated using correlation analyses.

**Results:**

In aquatic ecosystems, microbial cell density ranged from 1.8 × $10^{5}$ to 4.1 × $10^{6}$ cells mL^−1^ and was positively associated with specific conductance (*r* = 0.32, *p* < 0.01) and water temperature (*r* = 0.19, *p* = 0.03), but negatively associated with dissolved oxygen (*r* = −0.21, *p* = 0.01). Across all locations, the cell density averaged approximately 1.4 × $10^{6}$ cells mL^−1^. In terrestrial ecosystems, soil microbial biomass carbon ranged from 27 to 2.5 × $10^{4}$ μg C g^−1^ dry soil and was positively correlated with soil moisture (*r* = 0.57, *p* < 0.01), soil carbon content (*r* = 0.67, *p* < 0.01), and soil nitrogen content (*r* = 0.66, *p* < 0.01). It was negatively associated with soil temperature (*r* = −0.42, *p* < 0.01) and soil pH (*r* = −0.42, *p* < 0.01). Across all locations, the microbial biomass carbon averaged approximately 2.9 × $10^{3}$ μg C g^−1^. Bacteria were dominant across both aquatic and terrestrial environments, ranging from 28% to 88%, while Eukarya ranged from 0% to 48%. Archaea made a minor contribution to the microbial community across all sites. Unclassified microbes varied across the United States, ranging from less than 1% at the Lower Tombigbee River in southwest Alabama to 57% at Sycamore Creek in Arizona.

**Discussion:**

In aquatic systems, cell density increased with specific conductance and water temperature but decreased where dissolved oxygen was high. In terrestrial ecosystems, biomass was higher in soils with greater soil nitrogen content, soil carbon content, and moisture, indicating that nutrient-rich and humid environments favored microbial growth. In contrast, abundances declined in warmer and more alkaline soils. These biogeographic patterns show divergent environmental factors driving microbial abundance in various ecosystems, reflecting high microbial adaptation to surrounding physical and chemical conditions.

## 1 Introduction

In terrestrial ecosystems, soil microbes play a crucial role in the decomposition of organic matter, which facilitates the release of carbon (C), nitrogen (N), and phosphorus (P) and their utilization by plants in the soil (Likens, 1981; Filippelli, 2002; Poeplau et al., 2016; Zhao et al., 2024). The abilities of microbes to have processed organic material enabled lasting effects on N cycling and ecosystem functions (Delgado-Baquerizo et al., 2016). Microbes play vital roles in contributing to the health, balance, and sustainability of terrestrial environments. They have been key players in nutrient cycling, decomposition of organic matter, and recycling essential elements such as C, N, and P (Seymour and McLellan, 2025; Zaehle, 2013). In aquatic ecosystems, microbes help maintain water quality and support the base of the food web by providing nutrients for algae and other organisms (Hoshino et al, 2020). Additionally, certain microbes participate in both oxygen production and consumption, thereby influence the overall dynamics of aquatic ecosystems.

Microbes have been shown to follow biogeographic patterns (Xu et al., 2020; Martiny et al., 2006). Species interaction also shaped spatial patterns in microbes. There has been a dynamic relationship between microbes and other living organisms, which created spatial patterns in terrestrial and aquatic ecosystems (Cebrian and Lartigue, 2004; Soininen et al., 2015). Although microbial community composition has been widely examined, large-scale comparisons of microbial abundances across terrestrial and aquatic ecosystems remain understudied. Previous studies have compared microbial abundances across terrestrial and aquatic ecosystems through variables such as taxonomic diversity and genomic potential (Lozupone and Knight, 2007; Thompson et al., 2017). However, quantitative assessment of microbial biomass has provided distinct insights into the magnitude through physiochemical means, rather than the identity of microbial lineage (Whitman et al., 2018; Bahrain et al., 2018; Xu et al, 2013; Xu et al., 2014; Xu et al., 2017; He et al., 2020). Moreover, cross-ecosystem assessments are necessary to determine whether consistent environmental gradients influenced microbial abundance across different biomes (de Vries and Griffiths, 2018).

In this study, US National Ecological Observatory Network (NEON^1^) data were used to examine spatial variability in microbial biomass and cell density across terrestrial and aquatic ecosystems in the United States. We evaluated two primary hypotheses: (1) microbial abundance increases with greater nutrient and moisture availability in both environments, and (2) similar abiotic gradients, particularly those involving temperature, pH, and other various variables, govern microbial abundance across systems. The analysis focused exclusively on observed abundance-environment relationships, without inferring taxonomic composition or projecting climate effects.

## 2 Methodology and materials

### 2.1 Data collections

The data were derived from NEON, which provided standardized, open-access measurements of microbial biomass, microbial cell count, and concurrent environmental variables across continental scales (Thorpe et al., 2016; NEON (National Ecological Observatory Network), 2023). NEON’s sampled design and analytical protocols enabled robust comparisons among sites, spanning gradients in climate, vegetation, and substrate type. NEON has offered a unique opportunity to evaluate the physicochemical drivers that have influenced microbial abundance in both soils and surface waters. This study used publicly available datasets from the NEON (See footnote 1), which provided standardized, long-term ecological monitoring data collected across major US biomes. NEON conducts soil and aquatic samples, following consistent protocols that include measurements of microbial abundance, environmental chemistry, and physical parameters at each field site (Kao et al., 2012; Goodman et al., 2015; Qin et al., 2021).

Soil samples were collected periodically from terrestrial field plots to quantify microbial biomass and related physiochemical properties, while aquatic samples were collected from streams, rivers, and lakes to assess current abundance and community structure. These datasets span 2014 to 2023 and include measurements from multiple NEON domains that represent distinct climate and ecological regions across the United States (Supplementary Figure S1).

Cell density in aquatic ecosystems was quantified using cell counts (cells/mL) obtained by flow cytometry. Cell count samples in aquatic ecosystems were collected monthly, and 18 mL of water was preserved with 2 mL of 10% formaldehyde. Bacterial cells were stained with propidium iodine and counted under fluorescent microscopy at the Wyoming Stable Isotope Facility and the University of Florida Wetland Biogeochemical Lab. The study used data from NEON (2014–2023), including Water Community Microbes and Group Abundance, Soil Periodic, Benthic Community Microbes and Group Abundance, and Soil Microbial Community Count and Group Abundance (Supplementary Table S4). Prior to analysis, variables such as cell density, specific conductivity, water temperature, oxygen saturation, dissolved oxygen saturation, soil nitrogen content, microbial biomass, soil carbon content, soil moisture, soil pH, and soil temperature were converted by log transformation and then sequentially z-transformed to have met the Comparative Fit Index (CFI), Principal Component Analysis (PCA) and the Normed Fit Index models assumptions and compared variables (Supplementary Table S6).

### 2.2 Quantification of microbial abundance

**Figure 1:**
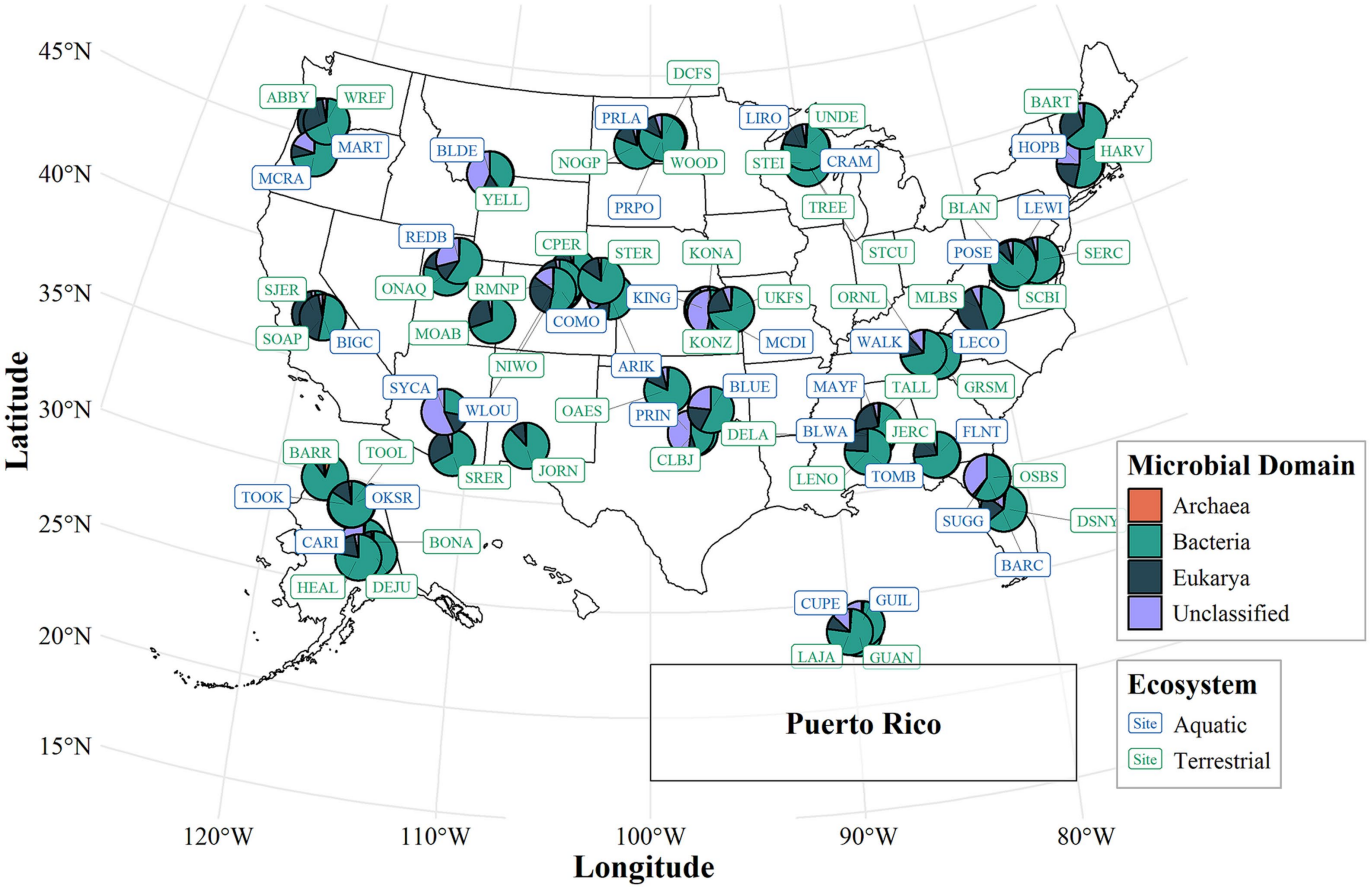
Site location and percentage of microbial abundance across the US.

To visualize the spatial scope of this study, a map represented 79 ecoclimatic domains that spanned major US biomes included arid deserts, temperate forests, tundra, and grasslands (Kao et al., 2012) (Figure 1 and Supplementary Figure S2). A site-only map distinguishing sampling locations, without abundance results, is provided in Supplementary Figure S2 and includes the corresponding site names for reference. Sampling locations are distributed across terrestrial and aquatic field sites within each domain, which represent diverse climate zones and vegetation types. Soil and water samples were collected at standardized NEON plots, and aquatic stations followed NEON’s protocols for acquisition of collected data, using site instrumentation recordings and microbial sampling techniques (Goodman et al., 2015; Qin et al., 2021).

In this study, the network’s 79 standardized domains encompassed a wide range of climatic, geographic, and ecological gradients, including deserts, forests, alpine regions, and tundra systems. This design made the data highly representative of the national environmental diversity, as described by Kao et al. (2012), who outlined how NEON’s ecoclimatic domains were created, and by Goodman et al. (2015), who documented the standardized field and laboratory protocols used across sites. Due to NEON’s uniform sampling procedures, automated quality control, and consistent instrumentation across all field locations, the dataset was considered a reliable benchmark for cross-biome ecological comparisons, as detailed in Qin et al. (2021). These standardized methods minimized regional bias and ensured that microbial abundance patterns reflected genuine spatial variation.

Additional environmental variables included measurements of soil and water temperature (°C), pH, soil moisture (g/g), N and C content (%), dissolved oxygen (mg/L), saturated oxygen levels (mg/L), specific conductance (μS/cm), soil fresh mass (grams), soil dry mass (grams), sample volume (ml), and biomass (μg C/g) (Goodman et al., 2015; Qin et al., 2021). The cell density and.

170 soil microbial biomass data was standardized following the equations:

$$C=C_{\mathrm{raw}}*\frac{\left(V_{s}+V_{p}\right)}{V_{s}}$$

$$L*f_{C}=B$$

where C represents the microbial cell density (cells $mL^{-1}$), $C_{\mathrm{raw}}\;$represents the raw microbial abundance reported by NEON (cells $mL^{-1}$), $V_{s}$ represents the original sample volume (mL), and $V_{p}\;$represents the preservative volume added to the sample (mL), B represents microbial biomass carbon (μg C $g^{-1}$ dry soil), Lrepresents the total lipid concentration (nmol $g^{-1}$), and $f_{C}$ represents the lipid-to carbon conversion factor (0.056 μg C $\mathrm{nmo}l^{-1}$).

### 2.3 Data processing and analysis

This study incorporated multiple NEON data products that together captured microbial and environmental variability across aquatic and terrestrial ecosystems from 2014 to 2023. In terms of terrestrial data, Soil Chemical Properties (DP1.10086.001) and Periodic (DP1.10078.001) provided data on soil temperature, soil moisture, pH, soil nitrogen percent, soil carbon percent, and biomass. In regard to aquatic data, Aquatic Microbe Cell Count (DP1.20138.001) provided dissolved oxygen, dissolved oxygen saturation, specific conductance, and water temperature measurements in aquatic ecosystems. Microbial abundance valued for aquatic and terrestrial data were provided through Benthic Microbe Group Abundances (DP1.20277.001), Benthic Microbe Community Composition (DP1.20086.001), Aquatic Microbe Community Composition (DP1.20141.001), and Soil Microbe Community Composition (DP1.10081.001), respectively. These datasets provided quantitative observations of microbial cell counts, group composition, and associated physiochemical properties across 79 NEON domains in the United States.

Statistical analysis was performed in an R script that examined spatial and environmental relationships influencing microbial abundances in aquatic and terrestrial ecosystems. Figure 1 was generated using the packages *ggplot2*, *sf*, *ggspatial*, and *scatterpie* to illustrate the spatial distribution and relative abundance of microbial domains (Archaea, Bacteria, Eukarya, and Unclassified taxa) across the 79 NEON domains. For each site, data were compiled by combining averages from the same season across multiple years, rather than combining data from different seasons to dampen within-seasonal variability, as well as to maintain consistency among biomes and minimize potential bias from uneven temporal samples. This approach provided a stable and representative summary of typical site conditions for large-scale spatial analyses, while acknowledgment of finer seasonal dynamics was beyond the scope of the present study.

**Figure 2:**
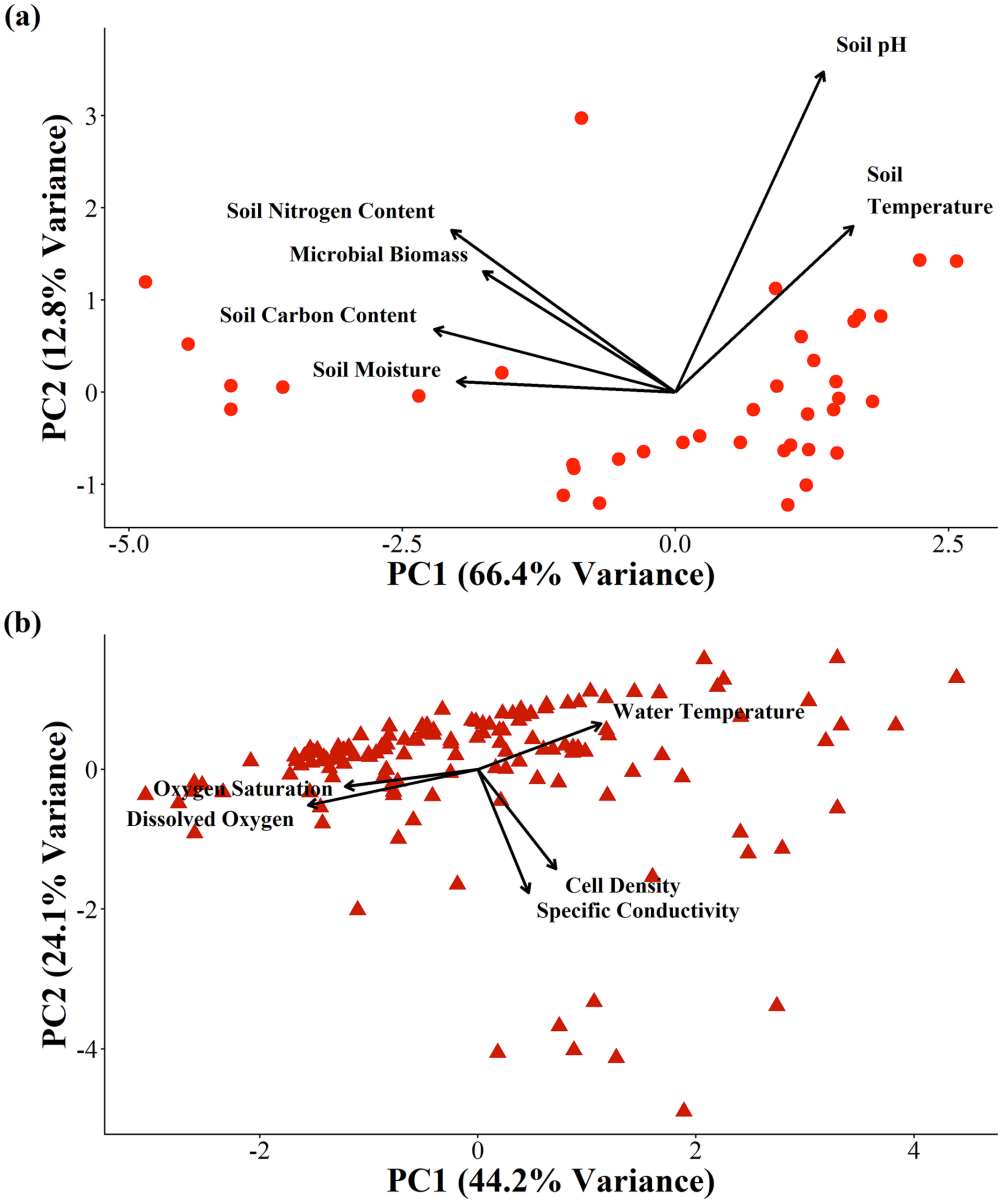
Principal component analysis of all factors in terrestrial ecosystems **(a)** and aquatic **(b)**.

Structural equation modeling (SEM) was carried out with the *lavaan* package; it was used to quantify the direct and indirect relationships between environmental factors and microbial abundance. Separate models were developed for aquatic and terrestrial ecosystems to represent specific interactions within these respective systems. The SEM models were defined *a priori* based on ecological theory for each ecosystem. In terrestrial systems, we assume that soil moisture regulates soil carbon content and soil nitrogen content availability, which act as the primary that direct drivers of microbial biomass, while soil temperature and pH function as secondary abiotic constraints that may indirectly influence nutrient accessibility. In aquatic systems, the model assumed that specific conductance and water temperature were the main physical and chemical drivers of microbial cell density, with dissolved oxygen and oxygen saturation included as mechanistically linked constraints on microbial metabolism. Variables were selected only if they were consistently measured across NEON sites, represented ecologically supported drivers of microbial abundance, and did not exhibit problematic collinearity. This priori structure ensured that the SEM pathways reflected meaningful, theory-based ecological relationships. In order to investigate the environmental variables impacts on microbial abundance, and created correlation without confounding bias, SEM Model fit was evaluated with the usage of the chi-square (χ^2^) goodness-of-fit test, the Comparative Fit Index (CFI), and the Normed Fit Index (NFI) to the variables associated with Figure 2 to assess how well the statistical model fit observed covariance patterns in the data and to quantify support for inferred relationships within the study.

Principal component analysis (PCA) was conducted to identify variables influencing microbial abundance across aquatic and terrestrial ecosystems. Prior to analysis, all environmental and microbial variables were log-transformed and then z-transformed to normalize data distribution and reduce the influence of extreme values. PCA was performed separately for aquatic and terrestrial datasets to assess the relative contribution of each environmental factor to overall variation in micro-abundance patterns. This procedure provided a means to visualize the quantification of the relationship among abiotic variables.

## 3 Results

### 3.1 Spatial patterns of microbial abundance across US ecosystems

Microbial biomass carbon varied substantially among NEON terrestrial sites, with values ranging from 2.5 to 27 × $10^{4}$ μg C g^−1^ of dry soil (Supplementary Table S1). Higher biomass levels were observed in northern NEON site regions of the continent, whereas lower values were found in the southwestern NEON site regions (Figure 1 and Supplementary Table S1). Across all sites, the average microbial biomass carbon was approximately 2.9 × $10^{3}$ μg C g^−1^, indicating substantial heterogeneity across biomes. Cell density in aquatic systems stayed roughly constant among NEON aquatic sites, with values ranging from 1.8 × $10^{5}$ to 4.1 × $10^{6}$ cells mL^−1^ (Supplementary Table S2). Across all sites, the average cell density was approximately 1.4 × $10^{6}$ cells mL^−1^.

Microbial community composition varied markedly across NEON sites in both terrestrial and aquatic ecosystems, highlighting their ecological versatility and abundance (Figure 1). Archaea accounted for 0–5% of the microbial community composition at each site. Bacteria dominated the microbial assemblages, accounting for 28–88% per site of total relative abundance (Supplementary Table S4). Eukaryotic microorganisms exhibited greater spatial heterogeneity, ranging from 0 to 48% per site, with higher relative abundance observed in humid, eastern, and southern regions. Unclassified taxa accounted for a notable component, ranging from 0 to 57% of total communities per site.

### 3.2 Correlation structure of microbial and environmental variables

**Figure 3:**
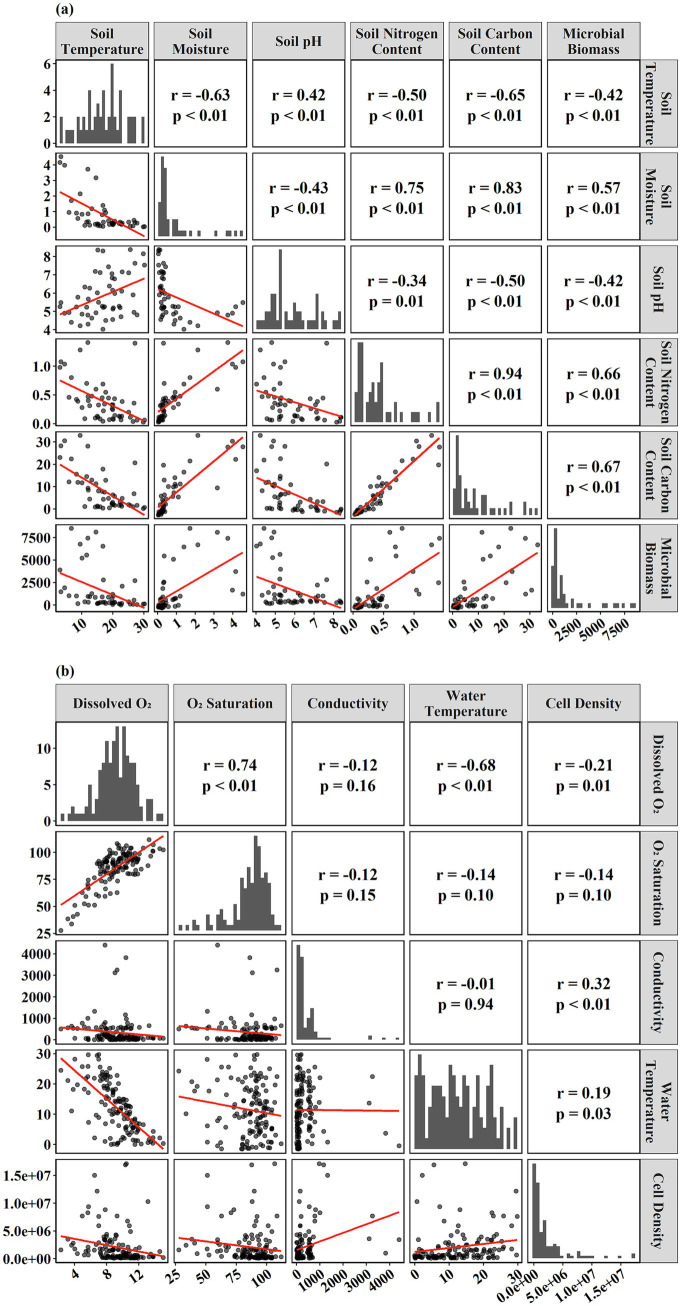
Correlation matrix of microbial abundance and environmental factors in soil **(a)** and aquatic ecosystems **(b)**.

In terrestrial ecosystems, pairwise correlations revealed that microbial biomass was significantly associated with multiple soil parameters (Figure 3a). Microbial biomass correlated positively with soil moisture (*r* = 0.57, *p* < 0.01), soil nitrogen content (*r* = 0.66, *p* < 0.01), and soil carbon content (*r* = 0.67, *p* < 0.01) and negativity correlated with soil temperature (*r* = −0.42, *p* < 0.01) and soil pH (*r* = −0.42, *p* < 0.01). Soil pH had significantly negative effects on soil carbon content (*r* = −0.50, *p* < 0.05) and soil moisture (*r* = −0.43, *p* < 0.05). Among the environmental variables themselves, soil temperature was negatively correlated with soil moisture (*r* = −0.63, *p* < 0.01), soil nitrogen content (*r* = −0.50, *p* < 0.01), and soil carbon content (*r* = −0.65, *p* < 0.01) positively correlated with soil pH (*r* = 0.42, *p* < 0.01). Soil nitrogen content and soil carbon content were strongly coupled (*r* = 0.94, *p* < 0.01). Soil nitrogen content exhibited a significantly positive effect on soil moisture (*r* = 0.75, *p* < 0.05) and a significantly negative effect on soil pH (*r* = −0.34, *p* < 0.01).

In aquatic ecosystems, microbial cell density varied in association with several water quality variables (Figure 3b). Cell density correlated negatively with dissolved oxygen (*r* = −0.21, *p* = 0.01) and correlated positively with specific conductance (*r* = 0.32, *p* < 0.01) and water temperature (*r* = 0.19, *p* = 0.03). Dissolved oxygen was positively correlated with oxygen saturation (*r* = 0.74, *p* < 0.01) and negatively correlated with water temperature (*r* = −0.68, *p* < 0.01). Oxygen saturation showed weak, non-significant relationships with specific conductivity (*r* = −0.12, *p* = 0.15) and temperature (*r* = −0.14, *p* = 0.10). Conductivity exhibited a weak positive relationship with cell density (*r* = 0.32, *p* < 0.01).

**Figure 4:**
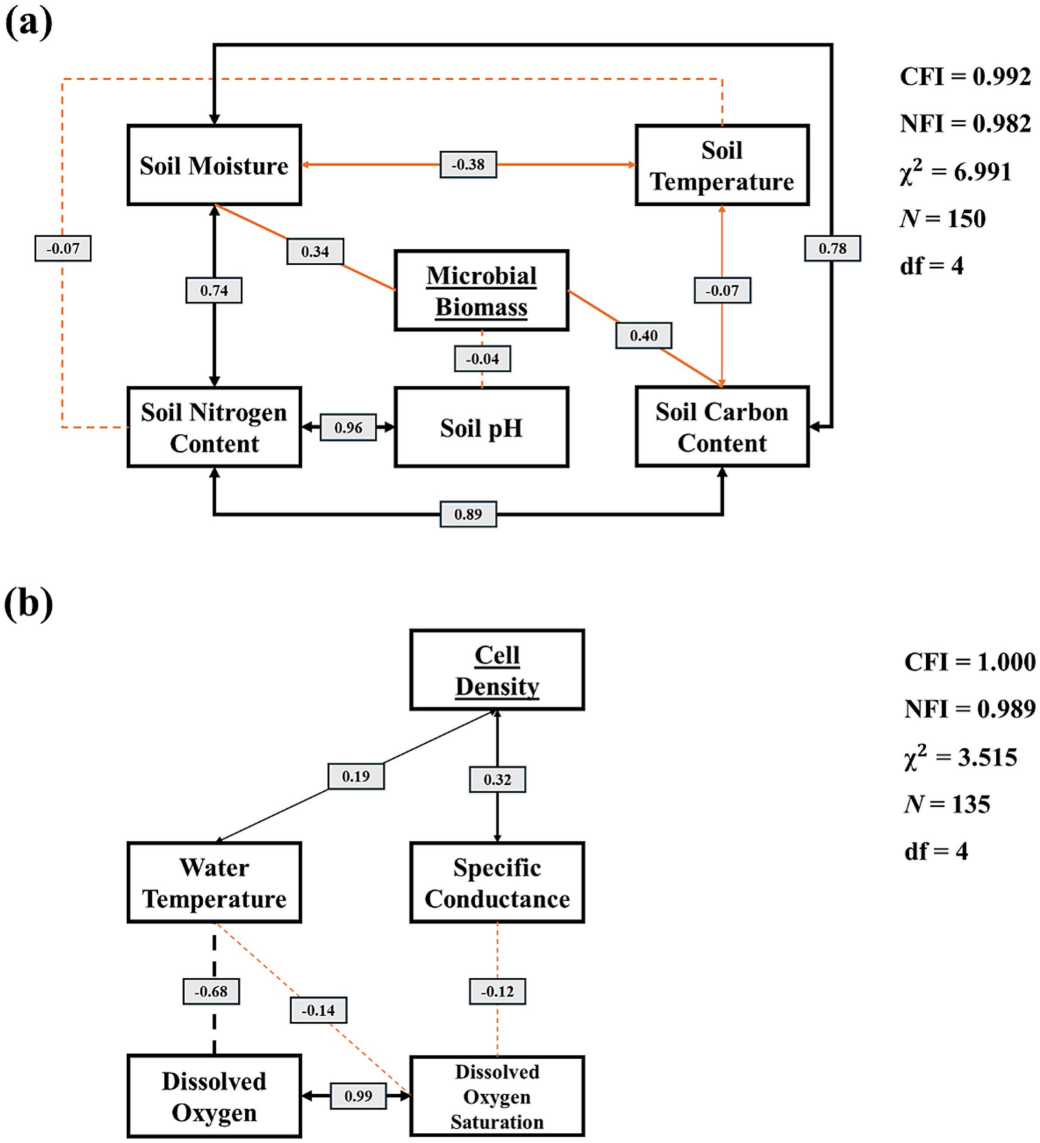
Structural equation model depicting microbial abundance and their environmental factors in terrestrial **(a)** and aquatic **(b)** ecosystems. Black lines represent significant effects [*p* < 0.05]; orange lines represent non-significant effects [*p* > 0.05]; solid lines represent positive paths; dotted lines represent negative paths. CFI, comparative fit index; NFI, normed fit index. (Terrestrial: CFI = 0.992, NFI = 0.982, χ^2^ = 6.991, df = 4, *p* > 0.05; Aquatic: CFI = 1.000, NFI = 0.989, χ^2^ = 3.515, df = 4, *p* > 0.05).

SEM captured the hierarchical relationships among environmental variables and microbial abundance in terrestrial and aquatic ecosystems (Figure 4a). Model fits were excellent for both systems. In terrestrial soils, soil moisture exerted a significant positive effect on soil nitrogen content (*r* = 0.74, *p* < 0.05). Soil moisture had a significantly positive effect on soil carbon content (*r* = 0.78, *p* < 0.05). Furthermore, soil carbon content had a significantly positive effect on soil nitrogen content (*r* = 0.89, *p* < 0.05) and soil nitrogen content had a significantly positive effect on soil pH (*r* = 0.96, *p* < 0.05).

In the aquatic ecosystems, specific conductance had a significant positive effect on microbial cell density (*r* = 0.32, *p* < 0.05), and water temperature exerted a smaller but significant positive influence (*r* = 0.19, *p* < 0.05) (Figure 4b). Dissolved oxygen and oxygen saturation were intricately linked (*r* = 0.99, *p* < 0.05). Water temperature exhibited significant negative paths to dissolved oxygen (*r* = −0.68, *p* < 0.05) and temperature exhibited an insignificant negative effect on oxygen saturation (*r* = −0.14, *p* > 0.05); conductivity showed a weak, non-significant negative connection to oxygen saturation (*r* = −0.12, *p* > 0.05).

PCA summarized the dominant environmental gradients in each ecosystem (Figure 2 and Supplementary Table S5). For terrestrial soils, the first two principal components explained 66.44 and 12.81% of total variance, respectively (Figure 2a). PC1 primarily represented variations in soil pH and temperature, whereas PC2 captured gradients in soil moisture, soil nitrogen content, soil carbon content, and microbial biomass. All the components together explained 99.9% of the total variance, which suggested the integrity of the data (Supplementary Table S5a).

For aquatic systems, PC1 and PC2 explained 44.16 and 24.12% of total variance of cell density, conductivity, and water temperature as well as dissolved oxygen and oxygen saturation with respect to PC1 and PC2 (Figure 2b). Together, the first five components accounted for more than 99% of the variance on the aquatic dataset in accordance with water temperature, cell density, specific conductivity, oxygen saturation, and dissolved oxygen saturation (Supplementary Table S5b). These component structures described the major axes of environmental variation without the implication causality.

## 4 Discussion

### 4.1 Microbial community distribution and spatial patterns

Microbial community composition varied widely across NEON sites, reflecting clear spatial and ecological structuring (Figure 1 and Supplementary Table S4). Bacteria dominated both terrestrial and aquatic ecosystems, accounting for most of the total abundance, while eukaryotic microorganisms became more heterogeneous, especially clustering in humid and coastal regions. Prokaryotes, such as bacteria, thrived in environments with specific temperature ranges, suitable pH levels, and nutrient availability, with varying oxygen requirements, depending on the species (Maier and Pepper, 2015). The higher representation of eukaryotic taxa near the West and East Coasts reflected the influence of marine or estuarine environments, including nutrient inputs, salinity gradients, and hydrologic connectivity (Cavicchioli et al., 2019). In contrast, inland terrestrial ecosystems were consistently dominated by bacterial groups adapted to fluctuations in temperature, soil moisture, and nutrient availability. The detection of unclassified taxa, especially in central US sites, suggested the presence of uncultured or poorly characterized microbial groups, and the need for improved molecular resolution (Suleiman et al., 2022). Collectively, these spatial patterns demonstrated strong biogeographic organization in microbial ecology with regional variation, driven by both environmental gradients and habit-specific selective pressures (He et al., 2023; Green and Bohannan, 2006).

Outliers were observed along the west and east coasts, where higher densities of eukaryotic microorganisms were found, suggesting a correlation with increased interactions with the ocean (Likens and Bormann, 1974). The high interaction with the ocean is due to the ocean boundaries reaching these distributions and abundances. This led to the conclusion that aquatic ecosystems played a dominant role in the dynamic established by both terrestrial and aquatic ecosystems (Likens and Bormann, 1974). To elaborate, in the central US, high-density spots of unclassified microorganisms were noted, possibly due to unculturable species, as a significant number of microorganisms cannot be cultured due to current technological limitations, which can explain the discrepancies and harsh data that lead to a skewed average (Suleiman et al., 2022).

In aquatic environments, particularly along the west coasts, a higher relative abundance of Eukarya was recorded (Figure 1). These may represent protists or other eukaryotic microbes, likely influenced by marine inputs, such as nutrient-rich coastal waters or estuarine mixing zones. This suggested that proximity to oceanic systems shifting community structure toward more eukaryotic-dominated assemblages, potentially due to differing energy sources, salinity gradients, or biotic interactions (Cavicchioli et al., 2019). In contrast, terrestrial ecosystems were characterized by a consistent dominance of bacterial groups, especially in drier, inland regions. These communities were shaped by soil texture, organic content, and temperature gradients, which were selected for highly adaptable bacterial taxa involved in processes such as decomposition and N cycling (Cebrian and Lartigue, 2004).

In the central US, several localized hotspots of unclassified microorganisms were detected (Figure 1), possibly representing incurable taxa or yet undescribed microbial lineages. The presence of these hotspots suggested either taxonomic data gaps or potentially novel microbial groups adapted to unique environmental niches. Spatial patterns also varied among study sites, reflecting regional differences in climate, vegetation, and soil chemistry (He et al., 2023). For example, microbial communities in forested soils of the northeast differed markedly from those in grasslands or desert biomes in the west. In comparison, aquatic samples from freshwater lakes contrasted sharply with estuarine or coastal zones in community composition and diversity. Together, these patterns demonstrated the complex biogeography of microbial communities across ecosystems, shaped by both environmental gradients and habitat-specific selective pressures.

### 4.2 Environmental drivers in aquatic ecosystems

Aquatic cell density was primarily shaped by specific conductance, water, temperature, and dissolved oxygen (Figures 2b, 4b and Supplementary Table S5b). Both conduct and temperature showed positive relationships with microbial cell density, indicating that ionic strength and thermal conditioning enhanced microbial activity and metabolic rates (Chapin et al., 2006). Specific conductance reflected the concentration of dissolved ions that supported microbial growth, while temperature influenced enzyme kinetics and nutrient turnover (Jin and Bethke, 2007; Price and Sowers, 2004). This interplayed among temperature, specific conductance, and oxygen availability highlights how physical and chemical gradients influenced microbial ecology and aquatic systems, contributed to spatial variation in carbon cycling and respiration dynamics (Nedwell, 1999).

A clear correlation is observed between specific conductance and water temperature, particularly in relation to microbial cell density, at various aquatic sites in the US. These factors were major contributors to the sustainability of microbial communities and were key elements, such as specific conductance and water temperature, in the C cycle (Chapin et al., 2006; Lin et al., 2016). Specific conductance influenced the C cycle through its effect on photosynthesis, as it indicated dissolved materials that microorganisms could use for growth, facilitating carbon uptake and its incorporation into the conveyor belt of aquatic ecosystem cycles (Chapin et al., 2006). Water temperature was equally critical, as it regulated the enzymatic activity and growth rates of microorganisms. Water temperature enhanced metabolic rates, accelerated the decomposition of organic matter and the carbon cycle through microbial respiration and biomass turnover (Chapin et al., 2006). However, excessively elevated temperatures could also lead to thermal stress, reducing microbial diversity and function. Importantly, temperature changes also have affected oxygen solubility, thereby influencing the balance between aerobic and anaerobic microbial processes (Nedwell, 1999).

### 4.3 Environmental drivers in terrestrial ecosystems

Although several variables were significantly correlated, only relationships with |*r*| ≥ 0.50 were considered strong. In terrestrial soils, microbial biomass was significantly associated with soil nitrogen content, soil carbon content, soil pH and soil moisture (Figure 3a). In contrast, warmer and more alkaline soils showed lower soil biomass, indicating sensitivity to temperature stress and pH imbalances (Singh and Gupta, 2018). Structural equation modeling revealed tightly coupled relationships among soil nitrogen, soil carbon, and soil moisture, which, in turn, indirectly drove soil biomass. While the direct effects of soil carbon content and soil moisture on biomass were positive, but statistically weak, their combined influences through nutrient interactions appeared to have regulated soil microbial dynamics. These interdependencies suggested that microbial abundance was controlled less by any single variable than by the integrated effects of nutrient and soil moisture availability, consistent with large-scale ecological observations (Kumar et al., 2022). High soil and microbial biomass might enhance ecosystem resilience, a crucial role in ecological restoration. This suggested that soil biomass was vital for the restoration and preservation of rational ecosystems, even when no apparent correlation with other variables was observed (Singh and Gupta, 2018).

### 4.4 Cross-ecosystem patterns and model interpretation

Although both ecosystems exhibited clear environmental controls on microbial abundance, the study’s objective was not to directly compare soil and aquatic systems, but to examine the internal relationships among environmental variables within each system (Figure 2). Aquatic abundance was best explained by specific conductance and temperature, whereas terrestrial abundance was driven by soil nitrogen content, soil carbon content, and soil moisture. The system highlighted the distinct ecological mechanisms that shaped micro- and macro-ecology across environmental gradients. Principal component analysis and structural equation modeling (Figure 2 and Supplementary Tables S5a,b) revealed that most variance in microbial abundance can be attributed to these key environmental axes. However, the current models primarily focus on large-scale spatial trends and do not explicitly account for potential site or seasonal effects (He et al., 2024). All variables were standardized using z-scores, which ensured comparability across sites; however, this normalization did not replace modeling random effects, such as location or sampling period (Supplementary Table S6).

### 4.5 Implications and future work

Previous research investigated the unique characteristics of terrestrial and aquatic ecosystems and their implications for sustainability, noted that each ecosystem possesses a distinct carbon footprint. In terrestrial systems, soil biomass, significant influence, biomass production, enzymatic activity, and carbon metabolism (Verrone et al., 2024). In aquatic environments, relative cell densities varied widely across habitat types (Stadler and del Giorgio, 2021). Future research should account for potential differences among sampling sites, as the current study focused primarily on environmental factors influencing soil composition and soil moisture. Incorporation of sites, specific variables, seasonal variation, and weather-related influences would provide a more comprehensive understanding of spatial and temporal patterns. Additionally, regional or state-level distinctions could enhance the interpretation of results and may have increased the study’s applicability. However, these patterns would require more comprehensive datasets and improved spatial sampling. Additionally, investigation of how temperature fluctuations affected microbial peaks illuminating the timing of key biological processes (Zhao et al., 2022; Zhou et al., 2020; Suleiman et al., 2022). While the study primarily relied on correlation analyses, PCA, and structural equation modeling identify environmental drivers of micro abundance, linear mixed models were not applied to control for potential site-specific or seasonal effects. This decision was based on the structure of the NEON dataset, which aggregated long-term, standardized measurements rather than continuous time-series observations at each site. Consequently, our analysis emphasized large-scale, spatial patterns, rather than temporal variations. Future work could incorporate hierarchical linear models into partition variance attributable to geographic location, climate domain, or sampling season (Jansson and Hofmockel, 2020). Such models would allow for a more precise quantification of the variance in microbial abundance explained by environmental factors, leading to a more refined explanation of the macro-ecological relationships identified here (Zhao et al., 2022; Patoine et al., 2022; He et al., 2024).

## 5 Conclusion

This research investigated continental-scale patterns in microbial abundance and the environmental factors that influence US soils and aquatic ecosystems, using NEON’s standardized datasets. By combining correlation, PCA, and structural equation modeling, this analysis provided one of the first nationwide assessments of microbial macroecology based on harmonized ecological data. This study demonstrated that microbial abundances were influenced by distinct environmental drivers in each ecosystem. In soil, microbial biomass was controlled by soil nitrogen and carbon content and soil moisture levels. In aquatic systems, cell density was driven by specific conductance and water temperature, highlighting distinct physicochemical controls that define microbial biogeography and cell density across US ecosystems.

## Data Availability

The original contributions presented in the study are included in the article/Supplementary material, further inquiries can be directed to the corresponding author/s. All data are obtained from the NEON data portal. The data and R scripts used in this study have been archived on GitHub, as mentioned in the main text.

## Footnotes

1
https://data.neonscience.org
