# Supplementary material for: Spatial biogeography of microbes in soils vs. aquatic ecosystems in U.S.’s major natural biomes
Source: Front Microbiol. 2026 Mar 12;17:1752205. doi: 10.3389/fmicb.2026.1752205 (PMC13018165; doi:10.3389/fmicb.2026.1752205)
Supplement: Supplementary file 1 [file Data_Sheet_1.DOCX]

Supplementary online material

for

**Spatial Biogeography of Microbes in Soils vs. Aquatic Ecosystems in U.S.’s Major Natural Biomes**

Leo Sai^1^, Xinhao Zhu^1^, David Lipson^1^, Xiaofeng Xu^1^

Biology Department, San Diego State University, San Diego, CA, USA

Corresponding author: X.X: [xxu@sdsu.edu](mailto:xxu@sdsu.edu); X.Z: xzhu2798@sdsu.edu

The file contains six tables and two figure

Table S1. Soil microbial biomass carbon in each study site.

| Study site | Microbial biomass [µg C/g] |
| --- | --- |
| ABBY | 1149.46104 |
| BARR | 1486.92432 |
| BART | 9751.34384 |
| BLAN | 445.2308 |
| BONA | 8943.55616 |
| CLBJ | 751.83416 |
| CPER | 428.31264 |
| DCFS | 1432.67096 |
| DEJU | 1038.75968 |
| DELA | 435.96056 |
| DSNY | 125.26584 |
| GRSM | 2595.12848 |
| GUAN | 4264.00072 |
| HARV | 17521.95424 |
| HEAL | 1925.3276 |
| JERC | 227.15616 |
| JORN | 270.44528 |
| KONA | 214.2476 |
| KONZ | 5705.8092 |
| LAJA | 631.44088 |
| LENO | 514.49272 |
| MLBS | 2371.66888 |
| MOAB | 865.35232 |
| NIWO | 7932.48848 |
| NOGP | 294.9464 |
| OAES | 111.5912 |
| ONAQ | 586.21584 |
| ORNL | 4597.90072 |
| OSBS | 1221.73128 |
| RMNP | 24675.82544 |
| SCBI | 555.93104 |
| SERC | 3444.3584 |
| SJER | 431.94536 |
| SOAP | 1190.3052 |
| SRER | 26.95224 |
| STEI | 376.87496 |
| STER | 2616.85592 |
| TALL | 1075.07344 |
| TOOL | 1731.72944 |
| TREE | 463.3608 |
| UKFS | 3916.04304 |
| UNDE | 545.034 |
| WOOD | 308.41888 |
| WREF | 13515.10272 |
| YELL | 2096.0548 |

Table S2. Microbial abundance in aquatic systems.

| Study site | Microbial abundance (cells /mL) |
| --- | --- |
| ARIK | 4064896.296 |
| BIGC | 1224279.194 |
| BLDE | 393726.1886 |
| BLUE | 340733.1973 |
| CARI | 3143195.053 |
| COMO | 4141588 |
| CUPE | 634860.4584 |
| GUIL | 344613.011 |
| HOPB | 1985795.618 |
| KING | 258249.0181 |
| LECO | 3311096.198 |
| LEWI | 1371544.861 |
| MART | 673859.4631 |
| MAYF | 1402665.312 |
| MCDI | 186573.2164 |
| MCRA | 1306390.824 |
| POSE | 1985045.891 |
| PRIN | 248242.6649 |
| REDB | 2309903.123 |
| WALK | 1042092.21 |
| WLOU | 203174.4054 |

**Table S3.** Pairwise t-test results comparing microbial domain abundances. Mean differences, t-statistics, degrees of freedom (df), and p-values are shown. All comparisons are statistically significant (*p* < 0.05), except for Eukarya vs. unclassified

| Microbial groups | Mean Difference | t-Statistic | df | p-Value |
| --- | --- | --- | --- | --- |
| Archaea vs Bacteria | -66.6013 | -45.9618 | 78.56258 | 1.52E-58 |
| Archaea vs Eukarya | -17.6709 | -13.415 | 78.68132 | 4.97E-22 |
| Archaea vs Unclassified | -13.4937 | -8.53116 | 78.47191 | 8.41E-13 |
| Bacteria vs Eukarya | 48.93038 | 25.03558 | 154.5913 | 2.90E-56 |
| Bacteria vs Unclassified | 53.1076 | 24.79815 | 154.8108 | 8.66E-56 |
| Eukarya vs Unclassified | 4.177215 | 2.033004 | 151.0204 | 0.043804 |

**Table S4.** Geographic distribution and relative abundance (%) of microbial domains (Archaea, Bacteria, Eukarya, and Unclassified) across NEON sites in the United States. Latitude and longitude coordinates are provided for each region

| Study site | Longitude | Latitude | Archaea [%] | Bacteria [%] | Eukarya [%] | Unclassified [%] |
| --- | --- | --- | --- | --- | --- | --- |
| ARIK | -102.447 | 39.75821 | 1 | 50.5 | 12.5 | 36 |
| BIGC | -119.258 | 37.05972 | 0.5 | 64 | 7 | 28.5 |
| BLUE | -96.6242 | 34.44422 | 0 | 57.5 | 19.5 | 23 |
| CARI | -147.504 | 65.15322 | 1.5 | 75.5 | 2.5 | 20.5 |
| COMO | -105.544 | 40.03496 | 1.5 | 49.5 | 33.5 | 15.5 |
| CUPE | -66.9868 | 18.11352 | 1 | 82 | 2 | 15 |
| GUIL | -66.7987 | 18.17406 | 1.5 | 70 | 2 | 26.5 |
| KING | -96.6038 | 39.10506 | 0.5 | 48.5 | 7 | 44 |
| LECO | -83.5038 | 35.69043 | 0 | 77 | 12.5 | 10.5 |
| LEWI | -77.9832 | 39.09564 | 0.5 | 40.5 | 18.5 | 40.5 |
| MART | -121.934 | 45.79084 | 0 | 55 | 15 | 30 |
| MAYF | -87.4077 | 32.96037 | 0.5 | 66.5 | 20.5 | 12.5 |
| MCDI | -96.443 | 38.94586 | 0 | 48 | 5 | 47 |
| MCRA | -122.166 | 44.2596 | 1 | 71 | 9 | 19 |
| POSE | -78.1473 | 38.89431 | 0.5 | 54 | 17 | 28.5 |
| STCU | -89.5864 | 45.50894 | 0 | 82 | 0 | 18 |
| SYCA | -111.508 | 33.75099 | 0 | 28 | 15 | 57 |
| WALK | -84.2793 | 35.95738 | 1 | 71 | 16.5 | 11.5 |
| WLOU | -105.915 | 39.89137 | 0.5 | 52.5 | 32 | 15 |
| ABBY | -122.33 | 45.76244 | 1 | 64 | 32 | 3 |
| BARR | -156.619 | 71.28241 | 5 | 85 | 8 | 2 |
| BART | -71.2874 | 44.06389 | 1 | 63 | 31 | 5 |
| BLAN | -78.0418 | 39.0337 | 0 | 87 | 9 | 4 |
| BONA | -147.503 | 65.15401 | 0 | 68 | 30 | 2 |
| CLBJ | -97.57 | 33.40123 | 0 | 79 | 16 | 5 |
| CPER | -104.746 | 40.81554 | 0 | 81 | 16 | 3 |
| DCFS | -99.1066 | 47.16165 | 0 | 73 | 21 | 6 |
| DEJU | -145.751 | 63.88112 | 0 | 57 | 41 | 2 |
| DELA | -87.8039 | 32.54173 | 1 | 72 | 18 | 9 |
| DSNY | -81.4362 | 28.12505 | 2 | 62 | 21 | 15 |
| GRSM | -83.502 | 35.68896 | 1 | 44 | 46 | 9 |
| GUAN | -66.8687 | 17.96955 | 0 | 61 | 25 | 14 |
| HARV | -72.1727 | 42.53691 | 1 | 63 | 33 | 3 |
| HEAL | -149.213 | 63.8758 | 0 | 78 | 19 | 3 |
| JERC | -84.4686 | 31.19484 | 0 | 73 | 23 | 4 |
| JORN | -106.843 | 32.59069 | 0 | 88 | 11 | 1 |
| KONA | -96.6129 | 39.11045 | 1 | 74 | 20 | 5 |
| KONZ | -96.5631 | 39.10077 | 1 | 78 | 15 | 6 |
| LAJA | -67.0769 | 18.02126 | 1 | 76 | 10 | 13 |
| LENO | -88.1612 | 31.85386 | 2 | 57 | 32 | 9 |
| MLBS | -80.5249 | 37.37831 | 1 | 44 | 48 | 7 |
| MOAB | -109.388 | 38.24828 | 0 | 70 | 27 | 3 |
| NIWO | -105.582 | 40.05425 | 0 | 65 | 32 | 3 |
| NOGP | -100.915 | 46.76972 | 1 | 80 | 15 | 4 |
| OAES | -99.0588 | 35.4106 | 1 | 81 | 14 | 4 |
| ORNL | -84.2826 | 35.96413 | 0 | 62 | 33 | 5 |
| OSBS | -81.9934 | 29.68928 | 1 | 59 | 26 | 14 |
| RMNP | -105.546 | 40.2759 | 0 | 56 | 41 | 3 |
| SCBI | -78.1395 | 38.89292 | 0 | 82 | 13 | 5 |
| SERC | -76.56 | 38.89013 | 0 | 82 | 14 | 4 |
| SJER | -119.732 | 37.10878 | 0 | 66 | 31 | 3 |
| SOAP | -119.262 | 37.03337 | 2 | 51 | 44 | 3 |
| STEI | -89.5864 | 45.50894 | 1 | 77 | 19 | 3 |
| STER | -103.029 | 40.46189 | 0 | 84 | 14 | 2 |
| TALL | -87.3933 | 32.95047 | 1 | 69 | 26 | 4 |
| TOOL | -149.37 | 68.66109 | 0 | 84 | 14 | 2 |
| TREE | -89.5857 | 45.49369 | 1 | 70 | 26 | 3 |
| UKFS | -95.1922 | 39.04043 | 1 | 72 | 21 | 6 |
| UNDE | -89.5373 | 46.23391 | 1 | 76 | 20 | 3 |
| WOOD | -99.2413 | 47.1282 | 0 | 82 | 13 | 5 |
| WREF | -121.952 | 45.82049 | 1 | 67 | 29 | 3 |
| YELL | -110.539 | 44.95348 | 1 | 72 | 25 | 2 |
| SRER | -110.835 | 31.91068 | 0 | 67 | 29 | 4 |
| BARC | -82.0084 | 29.67598 | 0 | 61 | 3 | 36 |
| BLDE | -110.587 | 44.95011 | 0 | 41 | 16 | 43 |
| BLWA | -87.7982 | 32.54153 | 0 | 74 | 0 | 26 |
| CRAM | -89.4737 | 46.20967 | 0 | 66 | 1 | 33 |
| FLNT | -84.4374 | 31.18542 | 0 | 83 | 7 | 10 |
| HOPB | -72.3295 | 42.47194 | 0.5 | 52.5 | 22 | 24.5 |
| LIRO | -89.7048 | 45.99827 | 0 | 66 | 1 | 33 |
| OKSR | -149.143 | 68.66975 | 1.5 | 81 | 3.5 | 14 |
| PRIN | -97.7823 | 33.37852 | 0 | 45 | 7 | 48 |
| PRLA | -99.1139 | 47.15909 | 0 | 75 | 0 | 25 |
| PRPO | -99.2532 | 47.12984 | 0 | 77 | 23 | 0 |
| REDB | -111.798 | 40.78393 | 0.5 | 59 | 11.5 | 29 |
| SUGG | -82.0178 | 29.68778 | 0 | 59 | 2 | 39 |
| TOMB | -88.1589 | 31.85343 | 0 | 76 | 24 | 0 |
| TOOK | -149.611 | 68.63069 | 0 | 67 | 3 | 30 |
| ONAQ | -112.452 | 40.1776 | 0 | 79 | 18 | 3 |

**Table S5.** Summary of principal component analysis (PCA) showing the percentage of variance explained by each principal component for terrestrial (Panel A) and aquatic (Panel B) datasets.

**(a)**

| **Terrestrial Principal Component** | **Terrestrial Variance Explained (%)** |
| --- | --- |
| **PC1** | 66.44 |
| **PC2** | 12.81 |
| **PC3** | 10.93 |
| **PC4** | 6.64 |
| **PC5** | 2.76 |
| **PC6** | 0.41 |

**(b)**

| **Aquatic Principal Component** | **Aquatic Variance Explained (%)** |
| --- | --- |
| **PC1** | 44.16 |
| **PC2** | 24.12 |
| **PC3** | 17.75 |
| **PC4** | 12.71 |
| **PC5** | 1.25 |

*
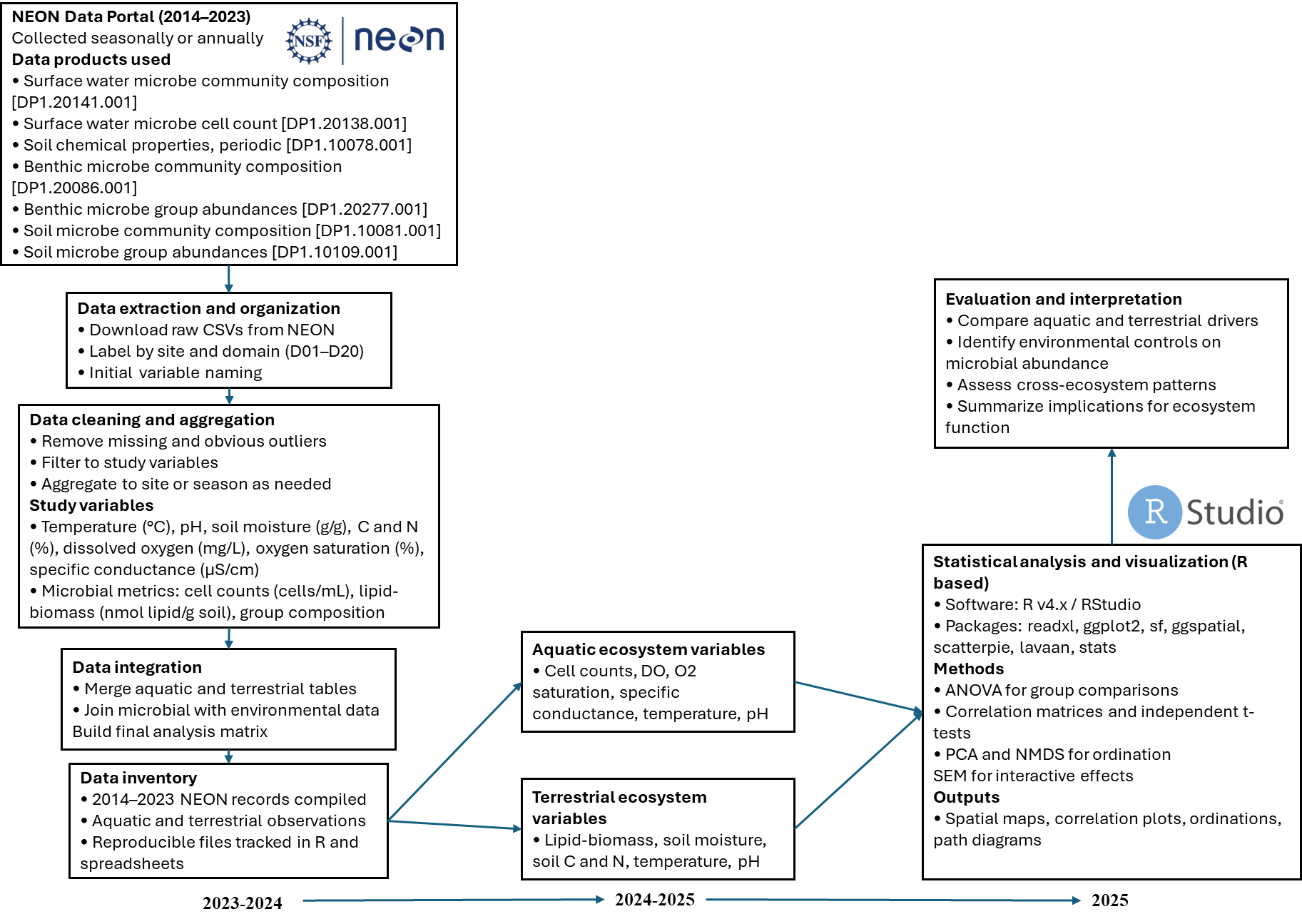
*

**Figure S1.** Analytical workflow outlining NEON data acquisition, data processing, and statistical analysis

**Table S6.** Summary of z-scores and corresponding p-values for structural relationships identified in the terrestrial (a) and aquatic (b) structural equation models. Positive z-scores indicate positive associations between dependent and independent variables, while negative z-scores represent inverse relationships. Significant pathways (p < 0.05) denote statistically supported effects among environmental and microbial factors.

**(a)**

| **Dependent Variable** | **Independent Variable** | **z- score** | **p-value** |
| --- | --- | --- | --- |
| Soil Carbon Content | Soil Temperature | -0.666 | 0.505 |
| Soil Carbon Content | Soil Moisture | 7.073 | p-value < 0.01 |
| Soil Nitrogen Content | Soil Temperature | 0.541 | 0.588 |
| Soil Nitrogen Content | Soil Moisture | 6.49 | p-value < 0.01 |
| Soil pH | Soil Carbon Content | -6.696 | p-value < 0.01 |
| Soil pH | Soil Nitrogen Content | 4.191 | p-value < 0.01 |
| Microbial Biomass | Soil pH | -0.625 | 0.532 |
| Microbial Biomass | Soil Carbon Content | 1.713 | 0.087 |
| Microbial Biomass | Soil Moisture | 1.255 | 0.209 |
| Soil Temperature | Soil Moisture | -4.245 | p-value < 0.01 |
| Soil Carbon Content | Soil Nitrogen Content | 2.872 | 0.004 |
| Soil Carbon Content | Soil Carbon Content | 3.517 | p-value < 0.01 |
| Soil Nitrogen Content | Soil Nitrogen Content | 2.628 | 0.009 |
| Soil pH | Soil pH | 9.064 | p-value < 0.01 |
| Microbial Biomass | Microbial Biomass | 4.209 | p-value < 0.01 |
| Soil Temperature | Soil Temperature | 10.294 | p-value < 0.01 |
| Soil Moisture | Soil Moisture | 4.281 | p-value < 0.01 |

**(b)**

| **Dependent Variable** | **Independent Variable** | **z- score** | **p-value** |
| --- | --- | --- | --- |
| Cell Density | Specific Conductance | 2.531 | 0.011 |
| Cell Density | Water Temperature | 2.151 | 0.032 |
| Dissolved Oxygen | Dissolved Oxygen Saturation | 5.866 | p-value < 0.01 |
| Water Temperature | Dissolved Oxygen Saturation | -1.576 | 0.115 |
| Dissolved Oxygen | Dissolved Oxygen | 6.777 | p-value < 0.01 |
| Specific Conductance | Specific Conductance | 2.402 | 0.016 |
| Cell Density | Cell Density | 3.452 | 0.001 |
| Water Temperature | Water Temperature | 11.315 | p-value < 0.01 |
| Dissolved Oxygen Saturation | Dissolved Oxygen Saturation | 5.843 | p-value < 0.01 |


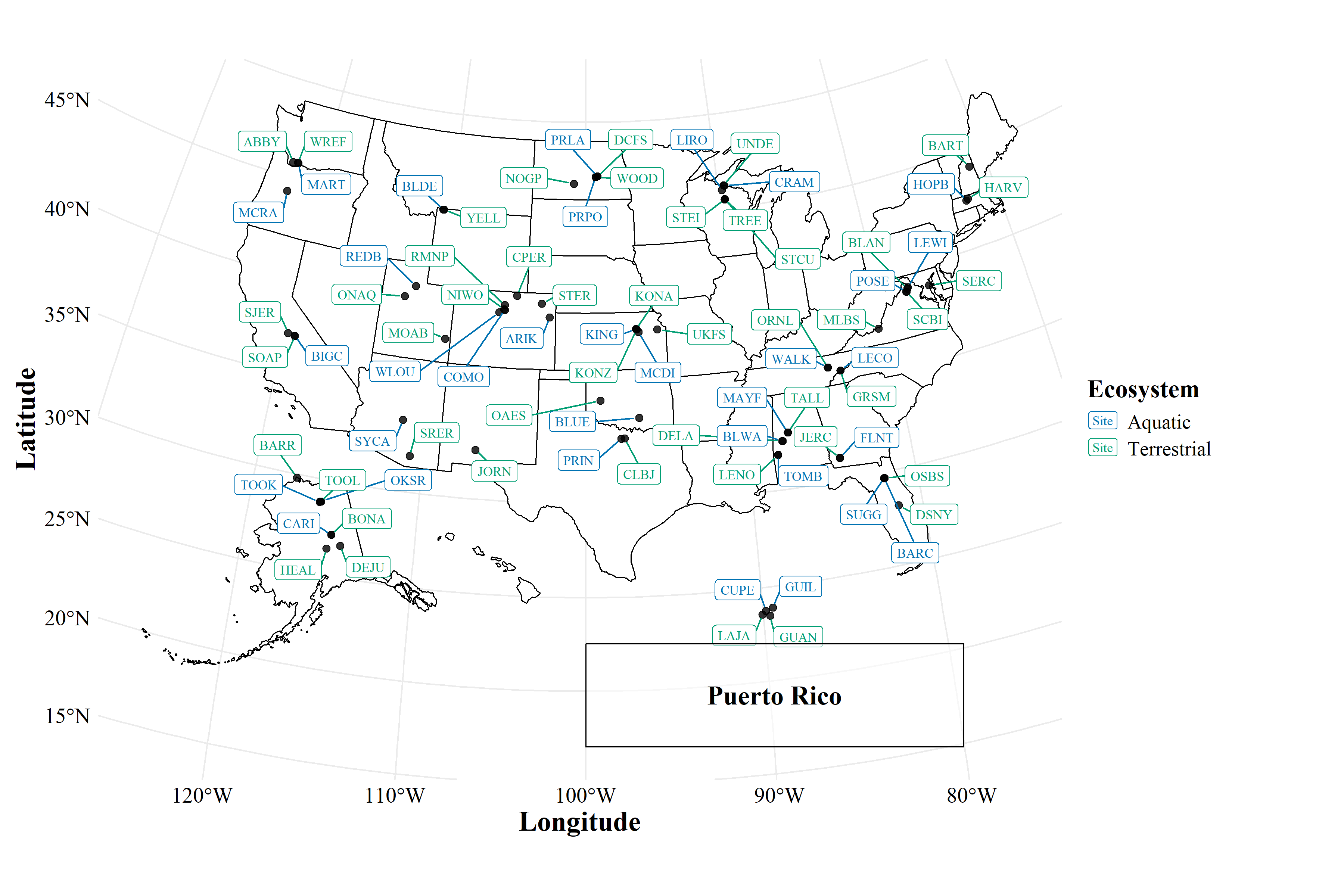


Figure S2. Site location across the US.
